# TIPE3 hypermethylation correlates with worse prognosis and promotes tumor progression in nasopharyngeal carcinoma

**DOI:** 10.1186/s13046-018-0881-5

**Published:** 2018-09-14

**Authors:** Xian-Yue Ren, Xin Wen, Ying-Qing Li, Jian Zhang, Qing-Mei He, Xiao-Jing Yang, Xin-Ran Tang, Ya-Qin Wang, Pan-Pan Zhang, Xiao-Zhong Chen, Bin Cheng, Jun Ma, Na Liu

**Affiliations:** 1 State Key Laboratory of Oncology in South China; Collaborative Innovation Center of Cancer Medicine; Guangdong Key Laboratory of Nasopharyngeal Carcinoma Diagnosis and Therapy, Sun Yat-sen University Cancer Center, 651 Dongfeng Road East, Guangzhou, 510060 People’s Republic of China; 2 Guangdong Provincial Key Laboratory of Stomatology, Guanghua School of Stomatology, Hospital of Stomatology, Sun Yat-sen University, Guangzhou, 510055 Guangdong People’s Republic of China; 3 Department of Radiation Oncology, Zhejiang Cancer Hospital, Hangzhou, 310022 Zhejiang People’s Republic of China

**Keywords:** TIPE3, Methylation, Prognosis, Proliferation, Metastasis

## Abstract

**Background:**

Increasing evidence recognizes that DNA methylation abnormalities play critical roles in cancer development. Our previous genome-wide methylation profile showed that tumor necrosis factor-alpha-induced protein 8 like 3 (TIPE3) was hypermethylated in nasopharyngeal carcinoma (NPC). However, the relationship between TIPE3 methylation and its mRNA expression, as well as its biological roles in NPC are unknown.

**Methods:**

Bisulfite pyrosequencing and quantitative RT-PCR were performed to quantify the TIPE3 methylation and expression levels. Kaplan-Meier curves and Cox regression analysis were used to estimate the correlation between TIPE3 methylation levels and survival in two patient cohorts collected from two hospitals (*n* = 441). The MTT, colony formation, Transwell migration and invasion assays, and xenograft tumor growth and lung metastatic colonization models were used to identify the functions of TIPE3 on NPC cells.

**Results:**

We found that TIPE3 CpG island (CGI) was hypermethylated and its mRNA levels were downregulated in many cancers, including NPC. TIPE3 downregulation was associated with its CGI hypermethylation. Furthermore, NPC patients with high TIPE3 CGI methylation levels had poorer clinical outcomes than those with low methylation levels. The TIPE3 CGI methylation level was an independent prognostic factor. Moreover, restoring TIPE3 expression significantly inhibited NPC cell proliferation, migration and invasion in vitro, and suppressed tumor growth and lung metastatic colonization in vivo, while silencing TIPE3 acted in an opposite way.

**Conclusions:**

TIPE3 downregulation correlates with its CGI hypermethylation in several solid cancers. TIPE3 acts as a tumor suppressor in NPC, providing a further insight into NPC progression and representing a potential prognostic biomarker for NPC.

**Electronic supplementary material:**

The online version of this article (10.1186/s13046-018-0881-5) contains supplementary material, which is available to authorized users.

## Background

Nasopharyngeal carcinoma (NPC) is a common head and neck cancer arising from the nasopharynx epithelium, with the highest prevalence in southern China [1, 2]. According to the National Comprehensive Cancer Network (NCCN) guideline, patients with early-stage disease should be treated with radiotherapy alone, while those with advanced-stage disease should receive combined chemoradiotherapy. The tumor-node-metastasis (TNM) staging system is the main determinant for prognostic prediction and treatment choices for NPC patients [3]. However, the TNM staging system cannot guide the best individualized treatment because of the biological heterogeneity exist among individuals. Approximately 20–30% of NPC patients eventually develop recurrence or distant metastasis [4]. During the past decades, great efforts have been made to better understand the molecular mechanisms involved in NPC progression. However, biomarkers that can help to accurately select patients with high risk of treatment failure remain absent. Thus, more studies are required to identify novel prognostic biomarkers to guide the individualized treatment for NPC patients.

DNA methylation, a representative epigenetic mechanism, can regulate gene expression through affecting the alternative promoters, retrotransposon elements, and other functional elements without changing the sequence of the nucleotides, which play important roles in the initiation and progression of cancer [5–7]. The dynamic nature of DNA methylation makes it reversible and can serve as an attractive target for cancer treatment [7, 8]. For the past decades, most studies on aberrantly DNA methylation in cancers focused on CpG islands (CGIs) in the promoter region of genes. CGI acquires hypermethylation to result in gene silencing, whereas DNA hypomethylation is linked to gene reactivation [5]. CGI hypermethylation has been recognized as one of the key features of cancer [9]. Recently, growing evidences indicate that there are high frequencies of CGI methylation of tumor suppressor genes in NPC, which contribute to the initiation and progression of NPC [10–13]. However, the roles of numerous aberrant methylation events in NPC are still unclear and warrant further studies.

TIPE family that contains a highly conserved seven α-helixes TIPE2 homology (TH) domain has been recognized as the regulators of inflammation and tumorigenesis. TIPE3 (also known as TNFAIP8L3) is a novel identified TIPE family member with a unique 19 amino acids N-terminal sequence, which has been reported as a transfer protein of lipid second messengers in regulating tumorigenesis [14]. Based on our previous genome-wide methylation microarray study (GSE52068) [12], we found that TIPE3 was significantly hypermethylated in NPC tissues in compared with the normal nasopharyngeal epithelial (NPEC) tissues. However, little is known about the effect of CGI hypermethylation on TIPE3 expression and the biological role of TIPE3 in NPC.

In this study, we analyzed the methylation status and mRNA expression levels of TIPE3 across all the solid cancer types in The Cancer Genome Atlas (TCGA) database and our own NPC tissues to identify the effect of TIPE3 CGI methylation on its transcription. Then, the relationships between the TIPE3 CGI methylation levels and clinical features of NPC patients in two large sample sets were analyzed. Furthermore, we investigated the effects of TIPE3 on NPC cell proliferation, migration, and invasion in vitro and in vivo, which may provide a more personalized therapy target for NPC patients.

## Methods

### Clinical specimens

25 freshly-frozen NPC biopsy samples and 21 normal nasopharyngeal epithelium tissues were collected from Sun Yat-sen University Cancer Center. In addition, a total of 441 formalin-fixed paraffin-embedded NPC biopsy tissue samples with detailed clinical follow-up information were obtained from Sun Yat-sen University Cancer Center (*n* = 254) and Zhejiang Cancer Hospital (*n* = 187) between 2004 and 2007. All samples were read and validated by two authoritative pathologists, and hematoxylin and eosin (H&E) staining confirmed that all of the slides contained > 70% tumor cells. All patients were restaged according to the 7th edition of the AJCC Cancer Staging System. No patents received any anti-tumor therapy before biopsy collection. Definitive radiotherapy was applied to all of the patients, and stages III-IV patients also received platinum-based concurrent chemotherapy. Regular clinical assessments were performed and the median follow-up time was 94 months (range: 2–139 months). This research was authorized by the Institutional Ethical Review Boards of both hospitals, and written informed consents were provided by all patients for using their biopsies.

### Cell culture and methyltransferase inhibitor treatment

All human immortalized NPEC cells and NPC cell lines were maintained in our own laboratory (Guangzhou, China). NPEC cell lines (NP69, N2-Tert, and N2-Bmi1) were maintained in Keratinocyte serum-free medium (Invitrogen) supplemented with bovine pituitary extract (BD Biosciences). NPC cell lines (CNE1, CNE2, SUNE1, HNE1 and HONE1) were grown in Roswell Park Memorial Institute (RPMI) 1640 (Invitrogen) supplemented with 10% fetal bovine serum (FBS) (Gibco). 293FT cells were maintained in Dulbecco’s modified Eagle’s medium (Invitrogen) supplemented with 10% FBS. For methyltransferase inhibitor treatment, 1.5 × 10^5^ cells were seeded on 60 mm culture dishes. After 24 h, the cells were cultured with or without the methyltransferase inhibitor 5-Aza-2′-deoxycytidine (DAC, 10 μM) for 72 h by replacing the drug every 24 h, and then harvested to extract DNA and RNA.

### DNA isolation and bisulfite pyrosequencing

An AllPrep RNA/DNA Mini Kit (Qiagen), QIAamp DNA FFPE Tissue Kit (Qiagen) or EZ1 DNA Tissue Kit (Qiagen) were used to extract the Genomic DNA from fresh-frozen tissues, FFPE tissues, or cell lines, according to the manufacturer’s instructions. An EpiTect Bisulfite Kit (Qiagen) was used to conduct the bisulfite modification of DNA (1-2 μg). The genomic region of TIPE3 chosen for bisulfite pyrosequencing was chosen according to our previous microarray data [15]. The bisulfite pyrosequencing primers were designed using the PyroMark Assay Design Software 2.0 (Qiagen). The primer sequences for PCR were as follows: 5′-GGGTTTGTAGGT TTATAGTTAATTT-3′ (forward); 5’-CCTCTCCCTAATACTAAACAACAA-3′(reverse); and for sequencing: 5′-TTGTGGGTAAGTGAGGA-3′. The sequencing reaction and methylation level quantification was conducted using the PyroMark Q96 ID System (Qiagen).

### RNA extraction and real time RT-PCR

Total RNA from NPC clinical specimens and cell lines was isolated using the TRIzol reagent (Invitrogen). Real-time RT-PCR was performed to test the mRNA expression levels of target gene as previously described [16]. Briefly, random primers (Promega) and M-MLV reverse transcriptase (Promega) were applied to synthesize the first strand cDNA. SYBR Green-based (Invitrogen) real-time PCR analysis was then performed using the CFX96 Touch™ sequence detection system (Bio-Rad). The primers for TIPE3 amplification were as follows: 5’-GATTGATGACA CCAGCACG-3′ (forward); 5’-TTTGATCGCCACCTTGAT-3′ (reverse). GAPDH was used as an endogenous control, and the comparative threshold cycle (2-ΔΔCT) equation was used to calculate the relative expression levels.

### RNA interference, plasmid construction and transfection

The sequence of HA-tagged human TIPE3 (NM_001311175.1) was cloned into plasmid pSin-EF2-puromycin between the EcoR I and Nhe I restriction sites. The pSin-EF2-TIPE3-HA and empty vector, as well as the lentivirus packaging plasmids psPAX2 and pMD2.G, were co-transfected into 293FT cells using the calcium phosphate method, as described previously [17]. After transfection, lentivirus particles were harvested and used to infect CNE2 and SUNE1 cells. The stably transfected cells were selected using puromycin (Sigma) and confirmed using western blotting. The small interfering RNAs for TIPE3 were purchased from GenePharma (Jiangsu, China), with the following sequences: siTIPE3–1, 5’-GAUGCCACGUUACAAACAATT-3′; siTIPE3–2, 5’-GACUUAAU CAAGGUGGCGATT-3′. CNE1 and HONE1 cells were transfected with siTIPE3s or control (100 nM) using LipofectamineTM RNAiMAX reagent (Invitrogen).

### Western blotting

Western blotting was performed to examine the protein levels of target genes, as previously described [16]. Radioimmunoprecipitation assay (RIPA) buffer containing a protease inhibitor cocktail (FDbio Science) was used for total protein extraction. Total proteins were separated using 10% sodium dodecyl sulfate-polyacrylamide gel electrophoresis, and then transferred to the polyvinylidene fluoride membranes (Millipore). After blocking in non-fat milk, the membranes were incubated with mouse monoclonal anti-HA antibody (1:1000; Sigma) at 4 °C overnight, followed by incubation with goat anti-rabbit secondary antibody (1:5000; Sigma). Finally, enhanced chemiluminescence (Thermo) was applied to test the antigen-antibody reaction, and GAPDH was used as a loading control.

### MTT and colony formation assays

For the MTT assay, 1 × 10^3^ cells were seeded per well in 96-well plates. After incubating for the indicated times, 20 μl of MTT (5 mg/mL, BD Biosciences) were added into each well, and then dimethylsulfoxide was used to resolve the crystals. The cell viability was recorded at 490 nm using a spectrophotometric plate reader. For the colony formation assay, 400 cells were plated per well in 6-well plates. After incubating for 7 or 12 days, the colonies were fixed with methyl alcohol, stained with 0.5% crystal violet, and then counted under an inverted microscope.

### Transwell migration and invasion assays

Transwell chambers with 8-μm pores in the membrane (Corning), pre-coated without or with Matrigel (BD Biosciences), were used to study the cell migration and invasion abilities. 5 × 10^4^ or 1 × 10^5^ cells suspended in serum-free medium were added into the upper chambers for migration or invasion assays, respectively, while medium supplemented with 10% FBS was placed into the lower chambers. After growing for 12 h (migration assay) or 24 h (invasion assay), the migrated or invaded cells were fixed with methyl alcohol, stained with 0.5% crystal violet, and counted under an inverted microscope (100×).

### In vivo xenograft tumor model

Animal experiments were approved by the Animal Care and Use Ethnic Committee of our Center, and all animal handling protocols were conducted based on the detailed principles to minimize animal suffering. 20 BALB/c nude mice (4 weeks old, female) were purchased from the Medical Experimental Animal Center of Guangdong Province (Guangzhou, China). For the xenografted tumor growth model, 1 × 10^6^ SUNE1 cells stably overexpressing TIPE3 or vector were injected into the right and left dorsal flank of mice (*n* = 5), respectively. After 7 days, the tumor size was measured every 3 days for 3 weeks. Then, the mice were sacrificed and the tumors were dissected and weighted. For the lung colonization model, 1 × 10^6^ SUNE1 cells stably overexpressing TIPE3 or vector were injected into the tail veins of mice (n = 5 in each group). After two months, the mice were sacrificed and their lungs removed. The lung tissues were fixed and paraffin-embedded before cutting into 5-μm slides. One of every ten slides was stained with hematoxylin and eosin (H&E) for microscopic observation.

### Statistical analysis

All statistical analyses were performed using Statistical Package for the Social Sciences 19.0 software (SPSS, Chicago, IL, USA), and a *p* value < 0.05 was considered as statistically significant based on two-sided tests. Data presented as the mean ± SD were calculated from at least three independent experiments. Continuous variables and categorical variables were compared using Student’s *t*-test, or chi-square and Fisher’s exact tests. The receiver operating characteristic (ROC) curve was used to identify the optimal cut-off value for high and low methylation of TIPE3. The Kaplan-Meier method and univariate Cox regression analysis were applied to estimate survival, and multivariate Cox regression analysis with the backward stepwise method was used to estimate independent prognostic factors. All data in our study have been recorded at Sun Yat-sen University Cancer Center for future reference (RDDB2018000414).

## Results

### TIPE3 CGI is hypermethylated in many human cancers

**Fig. 1:**
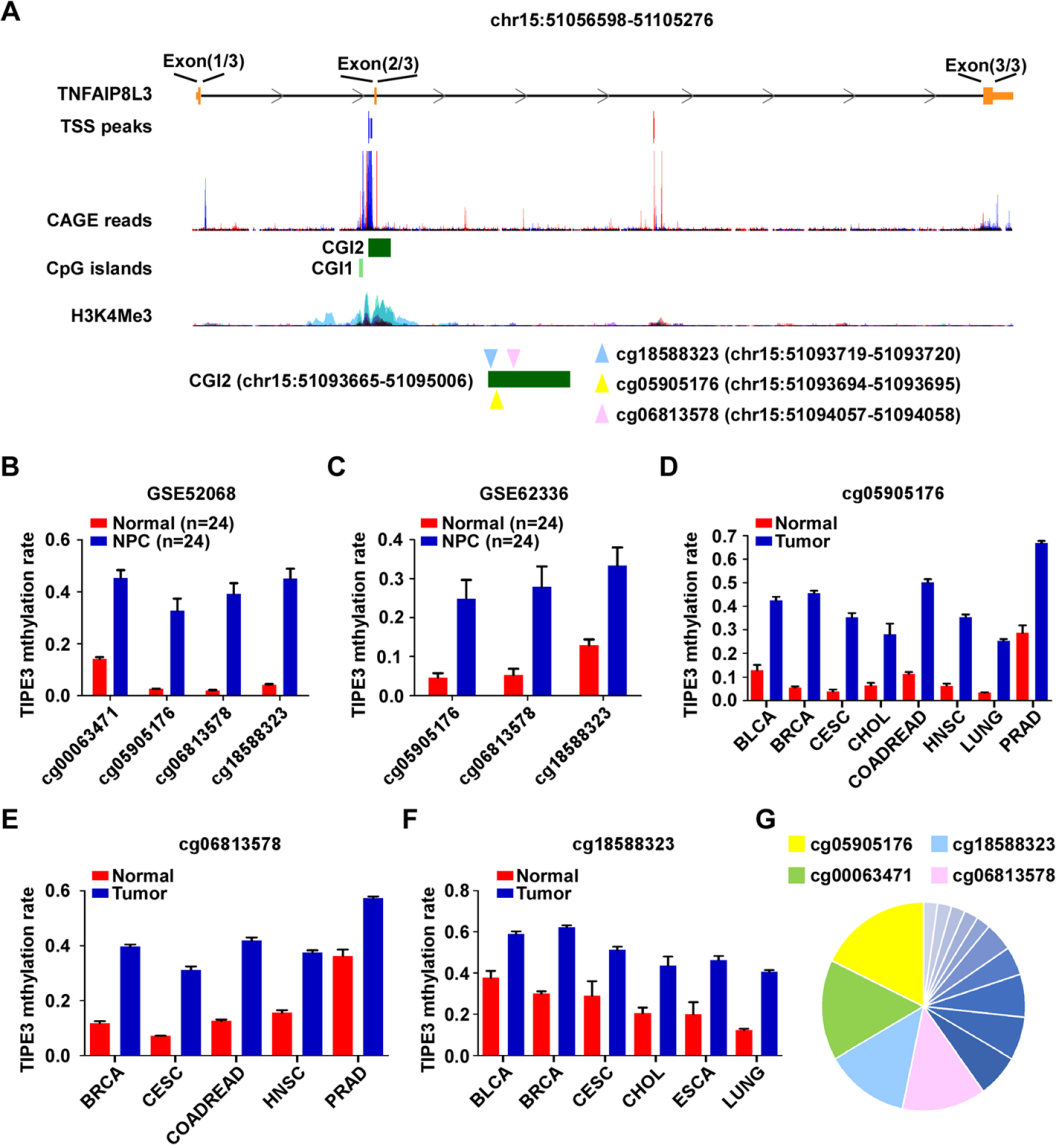
TIPE3 CGI is hypermethylated in several cancers. **a** The genome features of TIPE3 observed using the UCSC genome browser; **b** Relative methylation level of TIPE3 in NPC (*n* = 24) and NPEC tissues (n = 24), based on our previous microarray data (GSE52068); **c** Relative methylation level of TIPE3 in NPC (*n* = 25) and NPEC tissues (n = 25) from the HongKong microarray data (GSE62336); **d**-**f** The methylation levels of cg05905176 (**d**), cg06813578 (**e**), and cg18588323 (**f**) in cancers based on TCGA database; BLCA: bladder cancer; BRCA: breast cancer; CESC: cervical cancer; CHOL: cholangiocarcinoma; COADREAD: colon and rectal cancer; HNSC: head and neck cancer; LUNG: lung cancer; PRAD: prostate cancer; ESCA: esophageal carcinoma; **g** The proportion of aberrantly methylated TIPE3 CpG sites in pan-cancer. Mean ± SD, *p* < 0.05, Student’s *t*-test

We searched the UCSC Genome Browser and found two CGIs (CGI1, chr15:51093665–51,095,006; CGI2, chr15:51095336–51,095,558) located in the TIPE3 genome. Those CGIs showed strong H3K4me3 enrichment, a histone modification present at active promoters [17], in seven cell lines from ENCODE, suggesting that the CGIs of TIPE3 might function as alternative promoters. Furthermore, the CAGE (Cap Analysis Gene Expression) peaks, corresponding to the transcription start sites (TSSs) [15, 16], were identified within the CGIs of TIPE3 through the FANTOM project (Fig. 1a). Based on the Human Methylation 450 K BeadChip (Illumina), there are 28 CG probes within the TIPE3 genomic sequence (Additional file 1: Table S1). In our previous study (GSE52068), we identified four substantially hypermethylated probes for TIPE3 (cg05905176, cg00063471, cg18588323 and cg06813578) in NPC tissues compared to the NPEC tissues (Fig. 1b), which were confirmed by another published microarray data from Hong Kong (GSE62336 [15]), except for cg00063471 (Fig. 1c). The cg06813578, cg05905176 and cg18588323 were all located within the CGI2, which showed high H3K4me3 enrichment in TIPE3 genome (Fig. 1a).

Next, we examined the methylation levels of cg05905176, cg06813578, and cg18588323 in all types of solid cancer using the Human Methylation 450 K BeadChip (Illumina) data from TCGA database. The results showed that those CpG sites were significantly hypermethylated in different cancer types (Fig. 1d-f). Among all the differentially methylated CpG sites, cg05905176, cg00063471, cg18588323, and cg06813578 made up the major proportion (Fig. 1g), indicating that TIPE3 hypermethylation is a common event in human cancers.

**Fig. 2:**
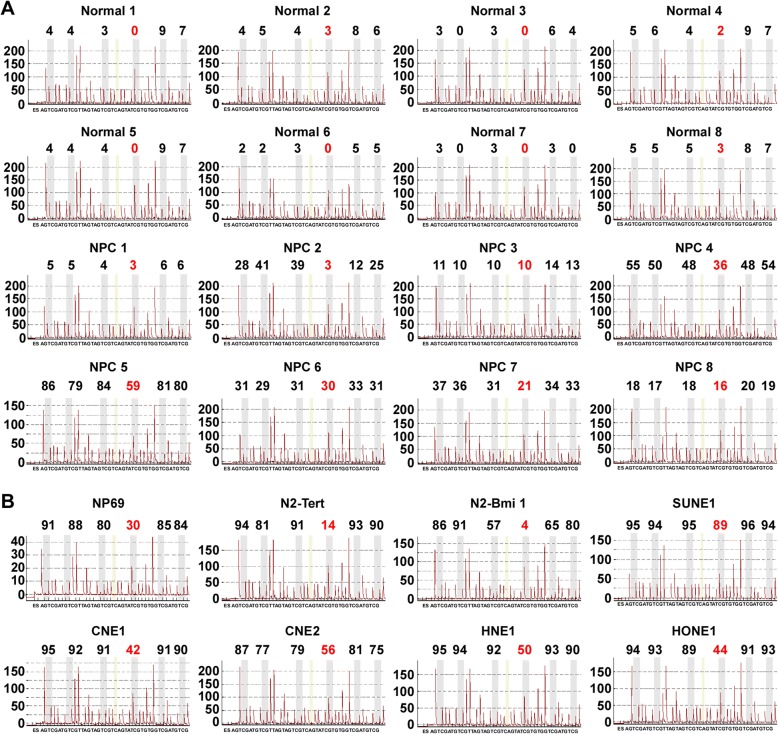
TIPE3 CGI is hypermethylated in NPC. **a** Bisulfite pyrosequencing analysis of the TIPE3 (cg05905176) methylation level in 8 NPC and 8 NPEC tissues; **b** Bisulfite pyrosequencing analysis of the TIPE3 (cg05905176) methylation level in NPEC cell lines (NP69, N2-Tert, and N2-Bmi1) and NPC cell lines (SUNE1, CNE1, CNE2, HNE1, and HONE1)

To verify whether the TIPE3 CGI was hypermethylated in NPC, we further tested the methylation levels of cg05905176 (the most significantly hypermethylated CpG site ranked by *p* value) in another eight NPC tissues and eight NPEC tissues, as well as five NPC cell lines and three NPEC cell lines using bisulfite pyrosequencing. The results showed that, compared with its level in NPEC, the methylation levels of cg05905176 were significantly increased in the NPC tissues (Fig. 2a) and cell lines (Fig. 2b).

### TIPE3 downregulation is associated with its CGI hypermethylation

**Fig. 3:**
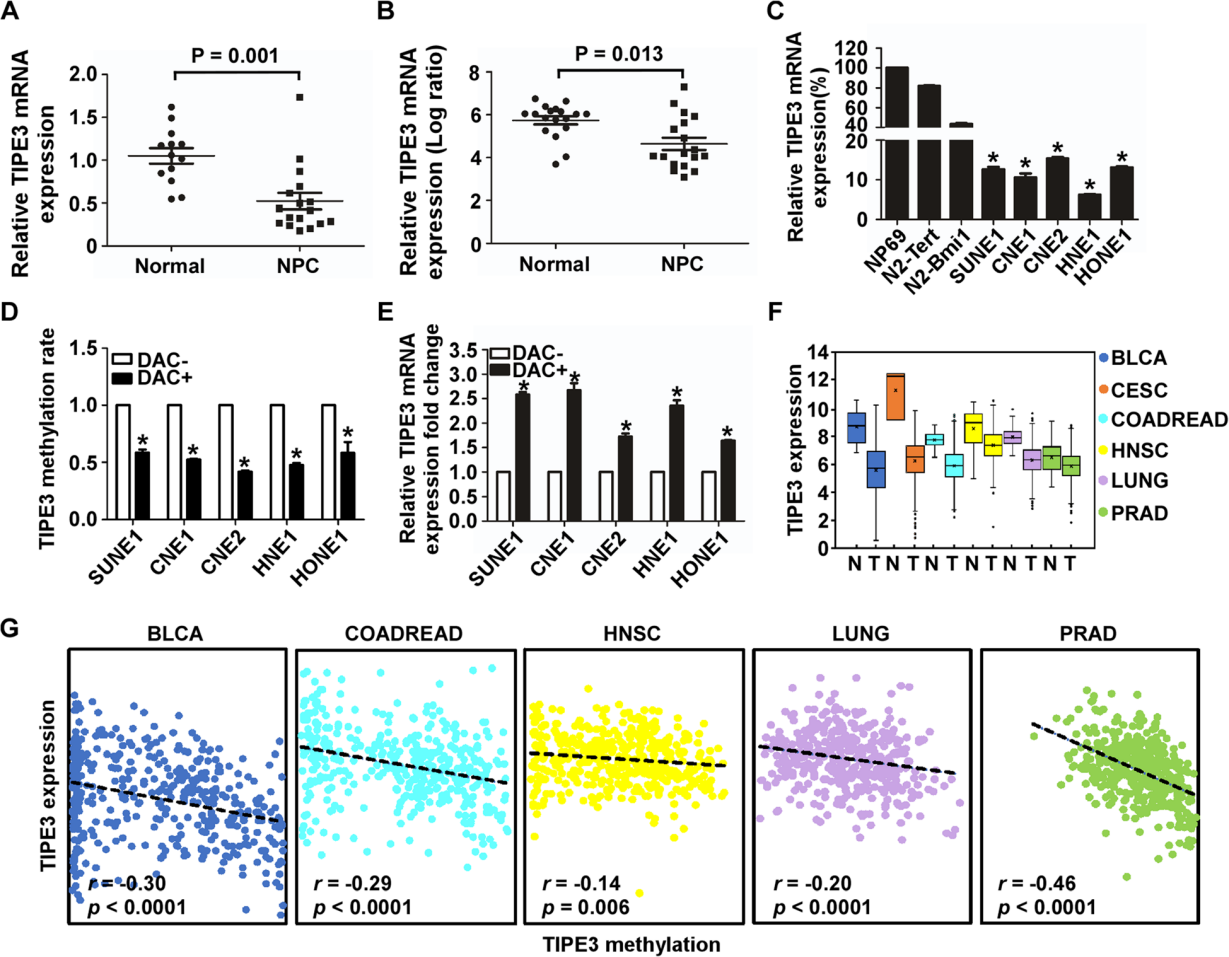
Downregulation of TIPE3 is associated with its CGI hypermethylation. **a** Relative mRNA level of TIPE3 in NPEC tissues (*n* = 13) and NPC (*n* = 17); **b** Relative mRNA level of TIPE3 in NPEC tissues (*n* = 18) and NPC (n = 18) from published microarray data (GSE53819); **c** Relative mRNA levels of TIPE3 in NPC and NPEC cell lines; **d**-**e** Relative methylation (**d**) and mRNA (**e**) levels of TIPE3 in NPC and NPEC cell lines treated with or without DAC; **f** TIPE3 mRNA level detected in cancers from TCGA database; T: tumor; N: normal; **g** The correlation between the TIPE3 (cg05905176) methylation level and mRNA level in cancers from TCGA database. Mean ± SD; *, *P* < 0.01, Student’s *t*-test

Next, we tested TIPE3 mRNA expression in NPC tissues (*n* = 17) and NPEC tissues (*n* = 13). We found that TIPE3 mRNA expression levels were obviously decreased in NPC tissues (Fig. 3a), which was verified using a published gene expression microarray data from the GEO database with 18 NPC tissues and 18 NPEC tissues (GSE53819 [17], Fig. 3b). Furthermore, we examined the TIPE3 mRNA levels in the NPEC and NPC cell lines using quantitative RT-PCR. As expected, the TIPE3 mRNA levels were significantly downregulated in NPC cell lines compared with those in the NPEC cell lines (Fig. 3c). To investigate the association between TIPE3 methylation status and its expression, the methylation and mRNA levels of TIPE3 were tested in NPC cells after treating with or without the demethylation drug DAC. After DAC treatment, the TIPE3 CGI methylation levels were significantly decreased (Fig. 3d), while the TIPE3 mRNA levels were increased in NPC cells (Fig. 3e).

**Fig. 4:**
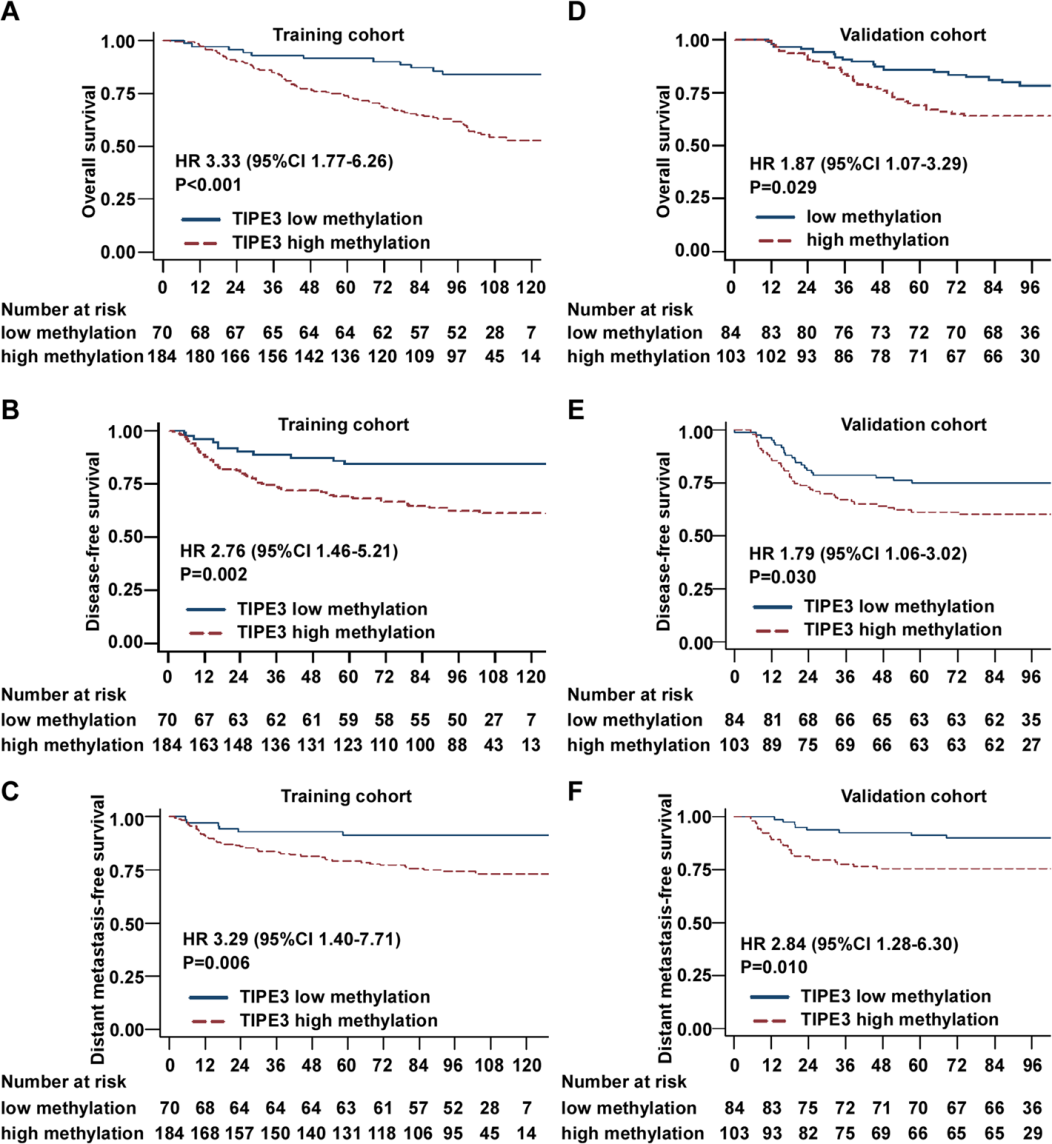
High TIPE3 CGI methylation level is associated with worse survival in NPC. **a**-**c** Overall survival (**a**), disease-free survival (**b**), and distant metastasis-free survival (**c**) in 254 NPC patients from the training cohort; **d**-**f** Overall survival (**d**), disease-free survival (**e**), and distant metastasis-free survival (**f**) in 187 NPC patients from the validation cohort. HRs and *P* values were calculated using univariate Cox regression analysis

We also investigated the TIPE3 mRNA expression across all types of solid cancer using the Illumina Hiseq data from TCGA database. We found that TIPE3 expression was significantly downregulated in six cancer types, including BLCA (bladder cancer), CESC (cervical cancer), COADREAD (colon and rectal cancer), HNSC (head and neck cancer), LUNG (lung cancer),and PRAD (prostate cancer) (Fig. 3f). To identify the relationship between the TIPE3 CGI methylation and mRNA levels, we performed Pearson’s correlation coefficient analysis using the same patients with both Illumina 450 K microarray data and Illumina Hiseq data. Except for CESC, the TIPE3 mRNA levels were found to be negatively related to the methylation levels of cg05905176 in five of the six cancers (Fig. 3g), implying that TIPE3 downregulation in human cancers is associated with its CGI hypermethylation.

### TIPE3 CGI hypermethylation was associated with worse survival in NPC

To determine whether TIPE3 CGI hypermethylation is associated with the clinical characteristics of NPC patients, we firstly tested the cg05905176 methylation levels using bisulfite pyrosequencing in 254 NPC samples of the training cohort (Sun Yat-sen University Cancer Center). Receiver operating characteristic (ROC) curve analysis was used to select the best cut-off value for TIPE3 low (< 3.5%) or high (≥ 3.5%) methylation groups. Among the patients,184 of 254 (72.4%) patients were classified into the high methylation group. As shown in Additional file 2: Table S2, no significant correlations were identified between TIPE3 methylation level and patients’ age, sex, WHO type, VCA-IgA, EA-IgA, T stage, N stage and TNM stage. Patients with high TIPE3 methylation levels had worse overall survival, disease-free survival (DFS), and distant metastasis-free survival (DMFS) than patients with low methylation levels (Fig. 4a-c).

To validate the prognostic value of the TIPE3 CGI methylation level, we determined the cg05905176 methylation levels using bisulfite pyrosequencing in another 187 NPC samples of the validation cohort (Zhejiang Cancer Hospital). We divided the patients into low or high TIPE3 methylation groups using the same cut-off value as used in the training cohort. The results showed that 103 of 187 (55.1%) NPC samples exhibited high methylation levels. We also found that there were no significant correlations between the TIPE3 methylation level and any of the clinical characteristics (Additional file 2: Table S2). Compared with patients with low methylation level, patients with high methylation level also had shorter OS, DFS, and DMFS (Fig. 4d-f).

### TIPE3 CGI methylation level is an independent prognostic factor in NPC

In addition, we performed univariate Cox regression analysis and discovered that the TIPE3 CGI methylation level and TNM stage were significantly associated with OS, DFS, and DMFS in both the training and validation cohorts (Additional file 3: Table S3). Furthermore, to explore whether the TIPE3 methylation level was an independent prognostic factor in NPC patients, we performed multivariate Cox regression analysis. The TIPE3 methylation level, age, sex, TNM stage, WHO type, VCA-IgA, and EA-IgA were used as covariates. We found that the TIPE3 CGI methylation level and TNM stage were independent prognostic factors associated with OS, DFS, and DMFS in both the training and validation cohorts (Table 1).

**Table 1:** Multivariate Cox regression analyses of the significant of different prognostic variables in nasopharyngeal carcinoma

	Overall survival			Disease-free survival			Distant metastasis-free survival		
Variable	HR	95%CI	*p-*value	HR	95%CI	*p-*value	HR	95%CI	*p-*value
Training set									
TIPE3 methylation level (High vs. Low)	2.99	1.59–5.64	0.001	2.56	1.35–4.85	0.004	2.91	1.24–6.28	0.014
TNM stage (III-IV vs. I-II)	2.00	1.13–3.54	0.018	1.86	1.03–3.38	0.041	2.68	1.14–6.28	0.023
Age (> 45 years vs. ≤45)	2.05	1.33–3.17	0.001	1.64	1.05–2.56	0.031	2.53	1.40–4.57	0.002
Validation set									
TIPE3 methylation level (High vs. Low)	1.80	1.02–3.16	0.041	1.71	1.01–2.89	0.045	2.69	1.21–5.96	0.015
TNM stage (III-IV vs. I-II)	3.30	1.41–7.71	0.006	3.19	1.45–7.01	0.004	3.71	1.13–12.18	0.030

### TIPE3 suppresses NPC cell proliferation, migration and invasion in vitro and in vivo

**Fig. 5:**
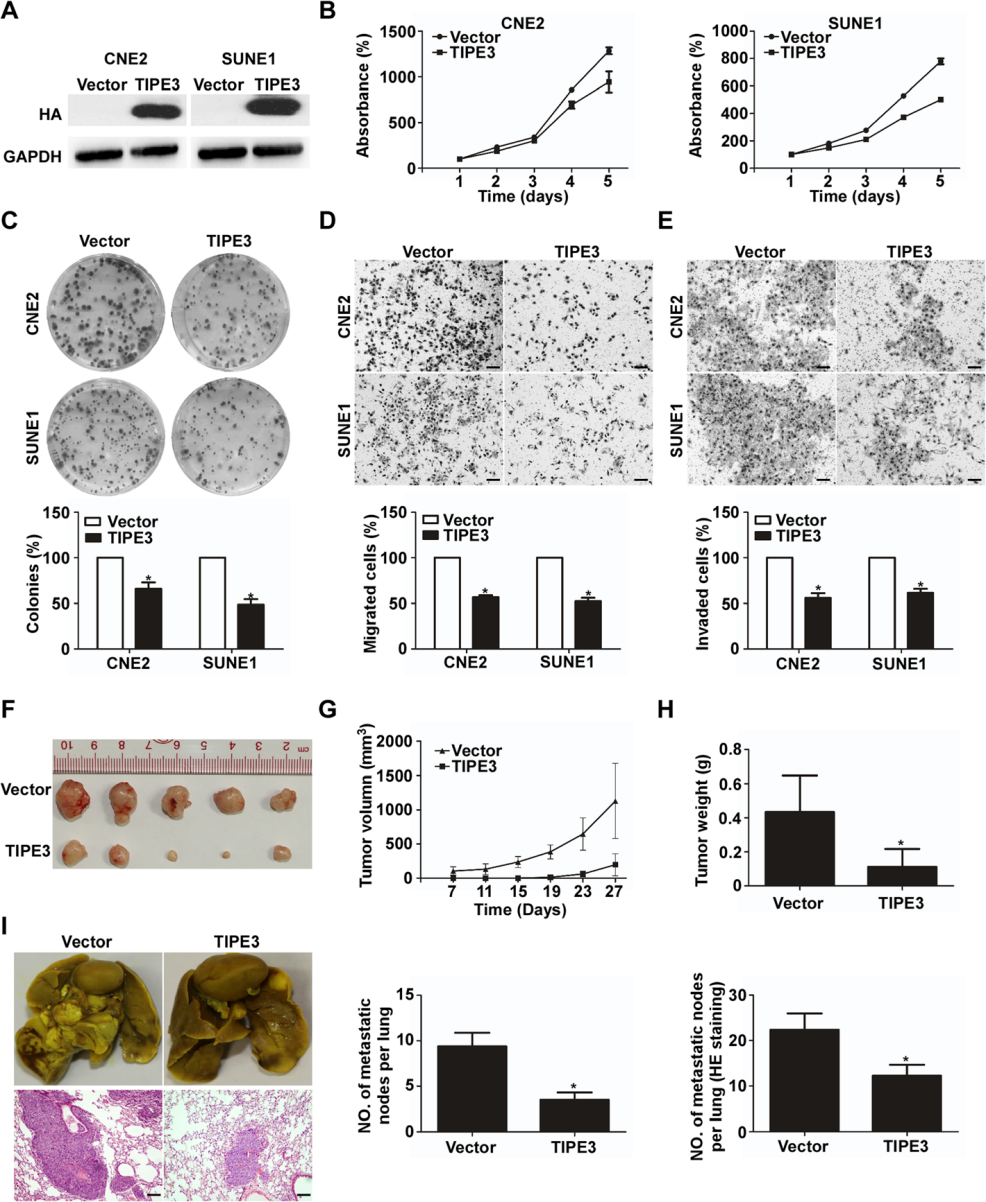
TIPE3 suppresses NPC cell progression *in vitro* and *in vivo.* **a** TIPE3 expression in CNE2 and SUNE1 cells stably overexpressing HA-tagged-TIPE3 or transfected with the empty vector; **b**-**e** Representative results of cell viability (**b**), colony formation (**c**), migration (**d**), and invasion (**e**) abilities of CNE2 and SUNE1 cells after stably overexpressing TIPE3 or vector; **f**-**h** SUNE1 cells stably overexpressing TIPE3 or vector were subcutaneously injected into the right and left dorsal flank of mice (n = 5); The tumor nodules (**f**), volumes (**g**), and weights (**h**) in mice xenografts; (**i**) SUNE1 cells stably overexpressing TIPE3 or vector was injected into the tail veins of mice (n = 5 in each group); Representative images and quantification of macroscopic tumor nodes on the surface of lung tissues and in lungs stained with H&E. Scale bar, 100 mm; Mean ± SD; *, *P* < 0.01

To investigate the roles of TIPE3 in NPC cells, we firstly constructed CNE2 and SUNE1 cells that stably overexpressed HA-tagged-TIPE3 or vector (Fig. 5a). MTT, colony formation, Transwell migration, and invasion assays were applied. Compared with the cells transfected with the empty vector, cells overexpressing TIPE3 showed suppressed cell viability (Fig. 5b), fewer colonies (Fig. 5c), inhibited migration (Fig. 5d) and invasive abilities (Fig. 5e).

To determine whether TIPE3 restoration affected NPC tumor growth and lung metastatic colonization in vivo, we constructed xenografted tumor growth and lung metastatic colonization models by injecting SUNE1 cells stably overexpressing TIPE3 or vector into the dorsal flank or tail vein of nude mice. In the tumor growth model, the mice were sacrificed for tumors after 27 days. Tumors formed in TIPE3 overexpression group had smaller volumes (Fig. 5f), slower growth rate (Fig. 5g), and lower weights (Fig. 5h) than those in vector group. In the lung metastatic colonization model, the mice were sacrificed for lungs after 2 months. Compared with mice injected with vector, the mice with tumors overexpressing TIPE3 formed fewer and smaller macroscopic metastatic nodes on the surfaces of their lungs, as well as microscopic metastatic nodes in their lungs determined by H&E staining (Fig. 5i).

**Fig. 6:**
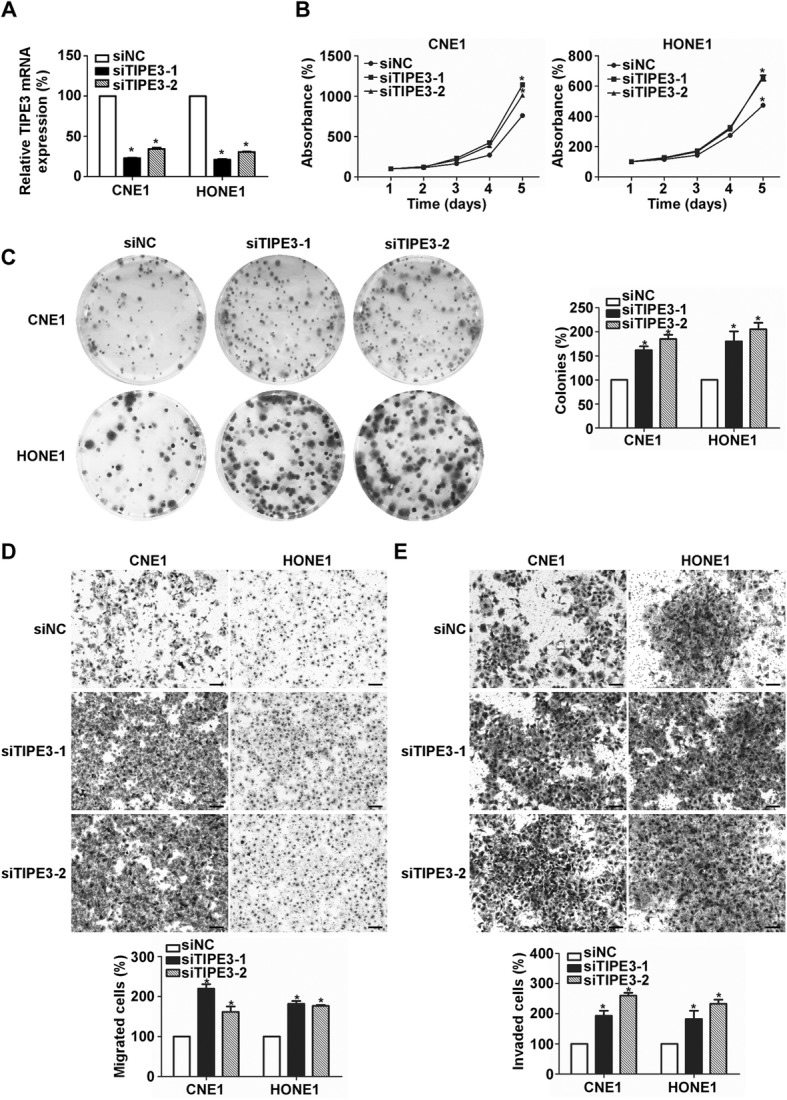
Silencing TIPE3 promotes NPC cell progression in vitro. **a** TIPE3 mRNA levels in CNE1 and HONE1 cells after transfection with TIPE3 siRNAs or control; **b**-**e** Representative results of cell viability (**b**), colony formation (**c**), migration (**d**), and invasion (**e**) abilities of CNE2 and SUNE1 cells after transfection with TIPE3 siRNAs or control. Scale bar, 100 mm; Mean ± SD; *, P < 0.01

At last, we knocked down the endogenous TIPE3 expression using two TIPE3 siRNAs in CNE1 and HONE1 cells (Fig. 6a). The results showed that suppressing TIPE3 expression could promote NPC cell proliferation (Fig. 6b-c), migration (Fig. 6d), and invasion (Fig. 6e).

## Discussion

Here, we found that the TIPE3 was hypermethylated and the mRNA was downregulated in several human cancers, including NPC. The downregulation of TIPE3 was associated with its CGI hypermethylation. Furthermore, NPC patients with high TIPE3 CGI methylation levels had worse clinical outcomes. Moreover, restoring TIPE3 expression suppressed NPC cell proliferation, migration and invasion in vitro, and inhibited tumor growth and lung metastatic colonization in vivo. Our findings suggested that TIPE3 hypermethylation has a potential to serve as prognostic biomarker and therapeutic target for NPC individualized therapy.

Epigenetic modifications play vital roles in regulating gene expression, such as DNA methylation, histone modifications, nucleosome positioning, and non-coding RNAs [5]. DNA methylation is among the best characterized epigenetic alterations, which usually occurs at CpG dinucleotides, where DNA methyltransferases catalyze the transfer of a methyl group to cytosine C-5 position to generate 5-methylcytosine. In mammals, genomic CpGs are enriched at CpG-rich short stretches known as CGIs, which are preferentially located at the promoters of genes [18]. Aberrant DNA methylations have been recognized involving in human cancer causation, progression and therapy [19, 20]. With the development of powerful technologies, the genome-scale DNA methylation maps of many cancers have been identified nowadays. Global DNA hypomethylation concomitantly with CGI hypermethylation have been identified in many cancers, like acute myeloid leukemia, colorectal cancer, and glioma [21–23]. In this study, through analyzing our previous methylation microarray data, numerous aberrant methylated CpG sites were found in NPC tissues, among which the TIPE3 was found to be remarkably hypermethylated, which was also observed in several human solid cancers in TCGA dataset, such as BRCA, HNSC, and CESC.

Gene expression is a complex process involving the packaging of DNA regulatory regions, chromatin modifying enzymes and transcription factors. Active gene promoters, especially those are CpG-rich and lacking DNA methylation, usually marked by H3K4me3, have extensive lysine acetylation and variant histone H2A.Z to facilitate transcriptional initiation [5]. Abnormal gains of DNA methylation in promoter CGIs induce gene transcriptional suppression, is a hallmark of human cancers. Here, we identified that TIPE3 expression was downregulated in NPC. The aberrant CGI methylation and expression state could be partially reversed by the demethylation drug in NPC cells. The TIPE3 mRNA level was inversely associated with its CGI methylation level. We also found that high levels of H3K4me3 enrichment, an epigenetic mark of active promoters, overlapping the TIPE3 CGIs in human cell lines from ENCODE. This suggests that this CGI can act as a promoter and further study will be necessary to determine the mechanisms by which it becomes methylated in NPC. Collectively, our results suggested that the downregulation of TIPE3 was related to its CGI hypermethylation, and epigenetic silencing of TIPE3 was a common event in human cancer.

The TIPE3 family consists of four members: TIPE, TIPE1, TIPE2, and TIPE3 [14, 24]. Recently, the TIPE family members have been recognized as inflammation, immunity, and cancer regulators [25, 26]. TIPE, the first identified member of this family, can regulate apoptosis and promote tumor metastasis and proliferation [27, 28]. TIPE1 was reported to be essential for TNF-α-induced cell death [29]. TIPE2 can maintain immune hemostasis and function as a tumor suppressor [30, 31]. TIPE3 was found to be upregulated in lung cancer, esophageal cancer, cervical cancer, and colon adenocarcinoma. The unique NT region of TIPE3, which is not seen in other members of the TIPE family, is believed to be responsible for its unique ability to promote cell growth and survival. Furthermore, TIPE3 lacking the NT region appeared to exert a tumor suppression effect [14]. In the present study, we found that ectopic expression of TIPE3 significantly suppressed NPC cell proliferation and invasion in vitro and in vivo, indicating that TIPE3 might act as a tumor suppressor in NPC and play a dual role in cancer progression. In fact, many genes are reported to act as either tumor suppressors or oncogenes in different cancer types. Nevertheless, the underlying mechanisms for these contradictory roles of TIPE3 in different cancers remain to be determined.

At present, the clinical decision making for NPC patients mainly relies on the TNM staging system [3], even though it cannot accurately select those at high risk of treatment failure. Over the past decades, numerous studies have focused on developing efficient prognostic molecular biomarkers, such as EBV-DNA, miRNAs, and gene expression [32–34]. However, there is still no effective predictive model for NPC patients. Recently, increasing evidences has demonstrated that both the single-gene loci and the genome-wide profiling indicated a strong potential to predict outcomes in malignant tumors. For single-gene loci, hypermethylation of CDKN2A in colorectal cancer, MGMT in glioblastoma, BRCA1 in breast cancer were reported to be associated with poor clinical outcomes [35–37]. For genome-wide profiling, the methylation gene panel as a prognostic biomarker of prostate, lung, and other cancers have also been identified [38, 39]. We previously constructed a six-hypermethylated gene panel to predict NPC patients’ survival [12]. However, the clinical applications of several aberrantly methylated genes in NPC remain unknown. In this study, our findings demonstrated that NPC patients with high TIPE3 CGI methylation level exhibited a significantly shorter OS, DFS, and DMFS compared with patients with low methylation level. These results implied that the TIPE3 CGI methylation level could help to identify a subgroup of patients with high risk of treatment failure and guide more individualized therapy.

## Conclusions

This study demonstrated that TIPE3 mRNA downregulation was correlated with its CGI hypermethylation in human solid cancers. NPC patients with low TIPE3 CGI methylation levels were at low risk of treatment failure, which might be caused by the tumor suppression effects of TIPE3. Therefore, TIPE3 is a potential novel prognostic biomarker and therapeutic target for NPC patients.

## Additional files

Additional file 1: **Table S1.** The methylation probes of TIPE3 in Infinium Human Methylation 450 K BeadChip (Illumina). (XLSX 16 kb)

Additional file 2: **Table S2.** Correlations between TIPE3 methylation levels and clinical features in patients with nasopharyngeal carcinoma from the training and validation cohorts. (DOCX 24 kb)

Additional file 3: **Table S3.** Univariate Cox regression analyses of the significant of different prognostic variables in nasopharyngeal carcinoma. (DOCX 19 kb)

## Abbreviations

AJCC: American joint committee on cancer
CGI: CpG island
CLL: Chronic lymphocytic leukemia.
DFS: Disease-free survival
DMFS: Distant metastasis-free survival
EA-IgA: Early antigen immunoglobulin A
EBV: Epstein-Barr virus
GAPDH: Glyceraldehyde-3-phosphate dehydrogenase
LDH: Lactate dehydrogenase
NCCN: National comprehensive cancer network
NPC: Nasopharyngeal carcinoma
NPEC: Normal nasopharyngeal epithelial cell
NT: N-terminal
OS: Overall survival
ROC: Receiver operating characteristic
RT-PCR: Reverse transcription-polymerase chain reaction
TCGA: The Cancer Genome Atlas
TIPE3: Tumor necrosis factor-alpha-induced protein 8 like 3
TNM: Tumor-node-metastasis
VCA-IgA: Viral capsid antigen immunoglobulin A
WHO: World health organization
