# Supplementary material for: TIPE3 hypermethylation correlates with worse prognosis and promotes tumor progression in nasopharyngeal carcinoma
Source: J Exp Clin Cancer Res. 2018 Sep 14;37:227. doi: 10.1186/s13046-018-0881-5 (PMC6137889; doi:10.1186/s13046-018-0881-5)
Supplement: Supplementary file 2 — Table S2. Correlations between TIPE3 methylation levels and clinical features in patients with nasopharyngeal carcinoma from the training and validation cohorts. (DOCX 24 kb) [file 13046_2018_881_MOESM2_ESM.docx]

**Table S2: Correlations between TIPE3 methylation levels and clinical features in patients with nasopharyngeal carcinoma from the training and validation cohorts**

|  | Training Cohort (n=254) | | P-value^*^ | Validation Cohort (n=187) | | P*-*value^*^ |
| --- | --- | --- | --- | --- | --- | --- |
| Characteristic | Low Methylation Group (%) | High Methylation Group (%) |  | Low Methylation Group (%) | High Methylation Group (%) |  |
|  | n=70 | n=184 |  | n=84 | n=103 |  |
| Age (mean ± SD) |  |  |  |  |  |  |
| ≤45 | 43 (61.4) | 92 (50.0) | 0.103 | 36 (42.9) | 41 (39.8) | 0.673 |
| >45 | 27 (38.6) | 92 (50.0) |  | 48 (57.1) | 62 (60.2) |  |
| Sex |  |  |  |  |  |  |
| Male | 49 (70.0) | 137(74.5) | 0.474 | 67 (79.8) | 69 (67.0) | 0.051 |
| Female | 21 (30.0) | 47(25.5) |  | 17 (20.2) | 34 (33.0) |  |
| WHO pathologic type |  |  |  |  |  |  |
| Undifferentiated non-keratinizing | 69 (98.6) | 180 (97.8) | 0.579 | 80 (95.2) | 93 (90.3) | 0.201 |
| Differentiated non-keratinizing | 1 (1.4) | 4(2.2) |  | 4(4.8) | 10 (9.7) |  |
| T Stage |  |  |  |  |  |  |
| T1-2 | 30 (42.9) | 67 (36.4) | 0.345 | 62 (73.8) | 67 (65.0) | 0.198 |
| T3-4 | 40 (57.1) | 117 (63.6) |  | 22 (26.2) | 36 (35.0) |  |
| N Stage |  |  |  |  |  |  |
| N0-1 | 45 (64.3) | 116 (63.0) | 0.854 | 37 (44.0) | 46 (44.7) | 0.933 |
| N2-3 | 25 (35.7) | 68 (37.0) |  | 47(56.0) | 57 (55.3) |  |
| TNM Stage |  |  |  |  |  |  |
| I- II | 21 (30.0) | 44 (23.9) | 0.321 | 25(29.8) | 23 (22.3) | 0.247 |
| III- IV | 49 (70.0) | 140 (76.1) |  | 59 (70.2) | 80 (77.7) |  |
| VCA-IgG |  |  |  |  |  |  |
| <80 | 8 (11.4) | 20 (10.9) | 0.899 | 59(70.2) | 68 (66.0) | 0.539 |
| ≥80 | 62 (88.6) | 164 (89.1) |  | 25 (29.8) | 35 (34.0) |  |
| EA-IgG |  |  |  |  |  |  |
| <10 | 14 (32.9) | 36 (23.3) | 0.938 | 17(20.2) | 31 (30.1) | 0.125 |
| ≥10 | 56 (67.1) | 148 (76.7) |  | 67 (79.8) | 72 (69.9) |  |
| Relapses or deaths |  |  |  |  |  |  |
| Yes | 11 (15.7) | 69 (37.5) | **0.001** | 21 (25.0) | 42 (40.8) | **0.023** |
| No | 59 (84.3) | 115 (62.5) |  | 63 (75.0) | 61 (59.2) |  |
| Distant metastasis |  |  |  |  |  |  |
| Yes | 6 (8.6) | 46 (25.0) | **0.004** | 8 (9.5) | 25 (24.3) | **0.009** |
| No | 64 (91.4) | 138 (75.0) |  | 76 (90.5) | 78 (75.7) |  |
| Death |  |  |  |  |  |  |
| Yes | 11 (15.7) | 80 (43.5) | **<0.001** | 18 (21.4) | 37 (35.9) | **0.030** |
| No | 59 (84.3) | 104 (56.5) |  | 66 (78.6) | 66 (64.1) |  |

*Chi-square test or Fisher’s exact test.
