# Supplementary material for: TIPE3 hypermethylation correlates with worse prognosis and promotes tumor progression in nasopharyngeal carcinoma
Source: J Exp Clin Cancer Res. 2018 Sep 14;37:227. doi: 10.1186/s13046-018-0881-5 (PMC6137889; doi:10.1186/s13046-018-0881-5)
Supplement: Supplementary file 3 — Table S3. Univariate Cox regression analyses of the significant of different prognostic variables in nasopharyngeal carcinoma. (DOCX 19 kb) [file 13046_2018_881_MOESM3_ESM.docx]

**Table S3: Univariate Cox regression analyses of the significant of different prognostic variables in nasopharyngeal carcinoma**

|  | **Overall survival** | | |  | **Disease-free survival** | | |  | **Distant metastasis-free survival** | | | |
| --- | --- | --- | --- | --- | --- | --- | --- | --- | --- | --- | --- | --- |
| **Variable** | **HR** | **95%CI** | **P*-*value** |  | **HR** | **95%CI** | **P*-*value** |  | **HR** | **95%CI** | **P*-*value** | |
| **Training set** | | | | | | | | | | | |  |
| TIPE3 methylation level (High vs. Low) | 3.33 | 1.77-6.26 | **<0.001** |  | 2.76 | 1.46-5.21 | **0.002** |  | **3.29** | **1.40-7.71** | **0.006** |  |
| TNM stage (III-IV vs. I-II) | 2.27 | 1.28-4.02 | **0.005** |  | 2.00 | 1.11-3.63 | **0.022** |  | 2.94 | 1.26-6.89 | **0.013** |  |
| Age (>45 years vs. ≤45) | 2.44 | 1.59-3.76 | **<0.001** |  | 1.80 | 1.15-2.81 | **0.010** |  | 1.87 | 0.91-3.83 | **0.089** |  |
| Sex (Man vs. Female) | 1.65 | 0.99-2.77 | 0.057 |  | 1.58 | 0.92-2.74 | 0.100 |  | 2.84 | 1.57-5.12 | **0.001** |  |
| WHO type (II vs. I) | 0.56 | 0.18-1.76 | 0.317 |  | 0.75 | 0.18-3.04 | 0.685 |  | 0.47 | 0.11-1.92 | 0.291 |  |
| VCA-IgG (≥80 vs. <80) | 2.48 | 1.00-6.11 | **0.049** |  | 2.08 | 0.84-5.14 | 0.113 |  | 2.18 | 0.68-7.00 | **0.190** |  |
| EA-IgG (≥10 vs. <10) | 1.47 | 0.83-2.60 | 0.187 |  | 1.21 | 0.68-2.16 | 0.510 |  | 1.39 | 0.65-2.95 | 0.392 |  |
| **Validation set** | | | | | | | | | | | |  |
| TIPE3 methylation level (High vs. Low) | 1.87 | 1.07-3.29 | **0.029** |  | 1.79 | 1.06-3.02 | **0.030** |  | 2.84 | 1.28-6.30 | **0.010** |  |
| TNM stage (III-IV vs. I-II) | 3.40 | 1.46-7.95 | **0.005** |  | 3.30 | 1.50-7.24 | **0.003** |  | 3.97 | 1.21-13.01 | **0.023** |  |
| Age (>45 years vs. ≤45) | 1.26 | 0.73-2.19 | 0.404 |  | 1.52 | 0.90-2.57 | 0.118 |  | 1.66 | 0.79-3.48 | 0.182 |  |
| Sex (Man vs. Female) | 1.16 | 0.63-2.13 | 0.630 |  | 1.29 | 0.72-2.30 | 0.397 |  | 1.21 | 0.55-2.68 | 0.639 |  |
| WHO type (II vs. I) | 0.66 | 0.28-1.55 | 0.341 |  | 0.96 | 0.38-2.40 | 0.929 |  | 0.77 | 0.24-2.53 | 0.670 |  |
| VCA-IgG (≥80 vs. <80) | 1.20 | 0.69-2.10 | 0.514 |  | 1.04 | 1.61-1.77 | 0.894 |  | 0.81 | 0.38-1.75 | 0.593 |  |
| EA-IgG (≥10 vs. <10) | 0.83 | 0.47-1.49 | 0.538 |  | 0.75 | 0.44-1.29 | 0.305 |  | 0.58 | 0.28-1.17 | 0.129 |  |
